# To Breathe or Not to Breathe: The Role of Oxygen in Bone Marrow-Derived Mesenchymal Stromal Cell Senescence

**DOI:** 10.1155/2021/8899756

**Published:** 2021-01-13

**Authors:** Dhir Niren Gala, Zsolt Fabian

**Affiliations:** ^1^School of Medicine, Faculty of Clinical and Biomedical Sciences, University of Central Lancashire, Preston, UK; ^2^Department of Medicinal Chemistry, Molecular Biology and Pathobiochemistry, Semmelweis University, Budapest, Hungary

## Abstract

Stem cell-based cellular therapy is a promising tool for the treatment of pathological conditions with underlying severe tissue damage or malfunction like in chronic cardiovascular, musculoskeletal, or inflammatory conditions. One of the biggest technical challenges of the use of natural stem cells, however, is the prevention of their premature senescence during therapeutical manipulations. Culturing stem cells under hypoxic conditions is believed to be a possible route to fulfill this goal. Here, we review current literature data on the effects of hypoxia on bone marrow-derived mesenchymal stromal cells, one of the most popular tools of practical cellular therapy, in the context of their senescence.

## 1. Introduction

Cellular therapy is a rapidly evolving field of regenerative medicine to regenerate damaged or injured tissues [1]. In order to fulfill the objectives of this therapeutic approach, the use of stem cells seems to be one of the most appropriate techniques due to their plasticity to give rise to terminally differentiated tissues. Their relative low abundance in source tissues, however, triggered attempts to expand them in vitro that raised various technical challenges including the prevention of their premature senescence.

### 1.1. Cellular Senescence

Cellular senescence, first described in fibroblast cultures, is a dynamic multistep process resulting in permanent proliferation arrest in response to various stimuli [2, 3]. At the molecular level, senescence is mediated by upregulation of the cyclin-dependent kinase inhibitor p16 and p21 with subsequent p53 stabilization [4]. Growth arrest is, eventually, accompanied by phenotypical hallmarks including dysregulation of the nuclear morphology, cytoplasmic inclusion of the chromatin, and the enlarged cytoplasm [5, 6]. The latter is characterized by enlarged, granular morphology, formation of actin stress fibers, mitochondrial accumulation, and lysosomal dysfunction and is, at least in part, mediated by the constitutive activation of the mTOR pathway [4]. At the transcriptional level, these phenotypic changes seem to be accompanied by fundamental alterations in gene expression patterns leading to upregulation of a number of signaling pathway that exert antiapoptotic effects [7–9]. In addition, the modified transcriptional pattern is also biased toward the robust expression of proinflammatory cytokines, chemokines, growth factors, and matrix-remodeling proteins culminating in the so-called senescence-associated secretory phenotype (SASP) [10]. Recent proteomic analysis of the SASP revealed that the SASP secretory profiles show high degree of heterogeneity among distinct cell types and senescence-inducing stimuli drawing an intricate picture of the senescence-specific transcriptional mechanisms [11]. Indeed, network analysis of the senescent transcriptome suggests upregulation of proteins like the hypoxia-inducible factor 1 (HIF1) as well as indicating the comprehensive nature of the changes of the senescent intracellular milieu [12]. Premature *ex vivo* senescence of stem cells prompted investigations on culturing methods including the use of differential oxygen tensions, exploiting the idea that low oxygen mimics one of the key aspects of the natural niche where these cells reside and, thus, help maintain critical phenotypic properties *in vitro*.

### 1.2. Mesenchymal Stromal Cells

Stem cells are typically grouped into two major classes: embryonic and adult stem cells. The former ones are long-known since cells in the inner cell mass of the blastocyst give rise to all the adult tissues ensuring physiologic organogenesis [13]. Adult stem cells are more tissue-specific and contribute to the remodeling of dedicated tissues only. They are found in various organs where their microenvironment critically affects their ability to differentiate [14]. One of these specialized niches is the bone marrow where adult stem cells are the most abundant in the form of hematopoietic and non-hemopoietic species [15]. The latter ones, termed bone marrow-derived mesenchymal stromal cells (BMSCs), were first identified by Friedenstein in 1976 [16]. BMSCs are multipotent species with the potential to differentiate into diverse mature cells in the mesenchymal lineages including adipo-, chondro-, and osteocytes, fibroblast, or, under certain conditions, other mesodermal cell types [17]. Additional characteristic features of BMSCs are that they are plastic-adherent in culture and express CD105, CD73, and CD90 surface markers but negative for CD45, CD34, CD14 or CD11b, CD79alpha, CD19, and HLA-DR [18]. At least in part due to the latter feature, BMSCs do not show alloreactivity in lymphocyte proliferative assays suggesting that they are suitable for transplantation between non-compatible HLA individuals [19]. Since human BMSCs (hBMSCs) have been shown to be able to differentiate into pericytes, myofibroblasts, osteocytes, and other mature cells contributing to the formation of the bone marrow microenvironment, they seem to play a role in regulating hemopoiesis by maintaining the hemopoietic microenvironment [20, 21] Indeed, transplantation of hBMSCs into the murine bone marrow was found to be sufficient to the reconstitution of the human bone marrow microenvironment and the formation of primitive human hemopoietic cells in the murine bone marrow [22]. It has also been well documented that, in humans, co-transplantation of hemopoietic precursors and hBMSCs improves hemopoietic recovery following therapeutic bone marrow transplantation [23, 24]. Besides their putative structural roles, hBMSCs maintain and develop the bone marrow microenvironment and support hemopoietic cells by producing various different cytokines as well [25]. In accordance, *in vivo* studies showed the constitutive expression of interleukin-1 and -6 (*IL-1*, *IL-6*, respectively), macrophage colony-stimulating factor (*M-CSF*), and stem cell factor (*SCF*) by hBMSCs differentiated towards the osteogenic lineage [26]. This, however, not only includes physiologic states but also occurs relevant in pathologic conditions. Indeed, the inflammatory bone marrow microenvironment of obese animals promotes mouse BMSC (mBMSC) differentiation into adipocytes [27]. These data suggest that hBMSCs are not only important factors in the maintenance of the bone marrow compartment but that microenvironmental stimuli might also be critical in their physiology including their reduced senescence that allows prolonged lifespan within their natural niche [28, 29]. BMSC senescence is a critical aspect of their use of cellular therapy since senescent BMSCs show differential proliferation and differentiation capacity [30]. Senescence is a particular challenge in BMSC-based cellular therapy due to the observations that senescent BMSCs also show reduced dynamics of microfilaments accompanied by impaired migration [31]. In addition, downregulation of numerous cytokine and chemokine receptors including the chemokine receptor type 4 (CXCR4) and chemokine receptor 7 (CCR7) is another characteristic finding in senescent BMSCs that might also contribute to the impaired migration of these cells, one of the critical aspects of their therapeutic potential [32]. One of the characteristic features of the bone marrow microenvironment is low oxygenation raising the question if local hypoxia is an essential microenvironmental factor of bone marrow-residing cells. Mathematical models predict that hemopoietic stem cells are localized to poorly vascularized bone marrow regions suggesting that the hypoxic microenvironment is essential for their homeostasis [33]. Indeed, consistent results were found in mice showing that hemopoietic cells reside in the hypoxic bone marrow niche [34]. Based on these data, one can speculate that local hypoxia in the bone marrow is an important microenvironmental factor in the physiology of BMSCs as well [35].

### 1.3. Hypoxia

Due to the fundamental role of oxygen in cellular physiology, oxygen depletion—hypoxia—is most often associated with pathologic conditions. Hypoxia, however, is an integral part of the development and maintenance of certain organs and tissues. Indeed, cytotrophoblasts proliferate in the 2% oxygen environment, whereas at 20% oxygen, they stop proliferating and undergo differentiation [36]. In accordance, the reproductive track of mammals is hypoxic, with O_2_ levels ranging from 1.5% to 8.7% until significant vascularization and formation of the placenta occurs towards the end of the first trimester [37, 38]. Thus, it is not surprising that embryonic stem cells have to be exposed to a hypoxic environment to maintain their pluripotency suggesting that oxygen affects intrinsic stem cell properties [39]. Interestingly, during syncytiotrophoblast formation, the expression of senescence markers was also observed raising the question of a potential link between the hypoxic adaptation and molecular machinery of senescence [40]. Hypoxia is characteristic not only of embryogenesis but also the physiology of adult organs, including the bone marrow raising the question if low oxygen tension has similar physiologic role of bone marrow-residing cells including hBMSCs than that of the emryonic stem cells. In support of this idea, hBMSCs were found to show higher proliferation rates in hypoxic conditions and remain undifferentiated at 2% oxygen levels indicating that hypoxia has complex effects on BMSCs [41].

### 1.4. Intracellular Response to Hypoxia

Cells in the low oxygen environment have intracellular signaling mechanisms that allow adaptation and response to reduced oxygen availability. The hypoxia-triggered complex molecular events are primarily orchestrated by the HIFs first identified as nuclear factors interacting with the 3′ enhancer sequence of the erythropoietin *(EPO)* gene in response to hypoxia [42]. It is made of two subunits: HIF-alpha (HIF*α*) and -beta (HIF*β*) of which the former one is responsive to oxygen levels while the latter is constitutively expressed [43]. In the presence of oxygen, HIF*α* is hydroxylated on conserved proline residues by prolyl hydroxylases (PHDs) [44]. This causes a conformation change in HIF*α* exposing a binding site for the von Hippel-Lindau (pVHL) ubiquitin ligase that polyubiquitylates hydroxylated HIF*α* for degradation [44, 45]. Since PHDs use molecular oxygen for the hydroxylation, they are considered to be the primary sensors of intracellular oxygen levels [46]. Indeed, in hypoxic conditions, hydroxylation and consequent degradation of HIF*α* fail leading to stabilization and dimerization of HIF*α* with the *β* subunit to upregulate the expression of multiple hypoxia-adaptive genes [47]. These include, for instance, the vascular endothelial growth factor (*VEGF*) that contributes to the survival of cells exposed to hypoxic conditions by facilitating angiogenesis [48]. The HIF-VEGF axis is functional in hypoxia-exposed hBMSCs suggesting that the unfold of their hypoxic phenotype is, at least in part, orchestrated by the HIF-mediated machinery [47].

### 1.5. Molecular Characteristics of BMSCs in the Oxygen-Depleted Environments

**Figure 1:**
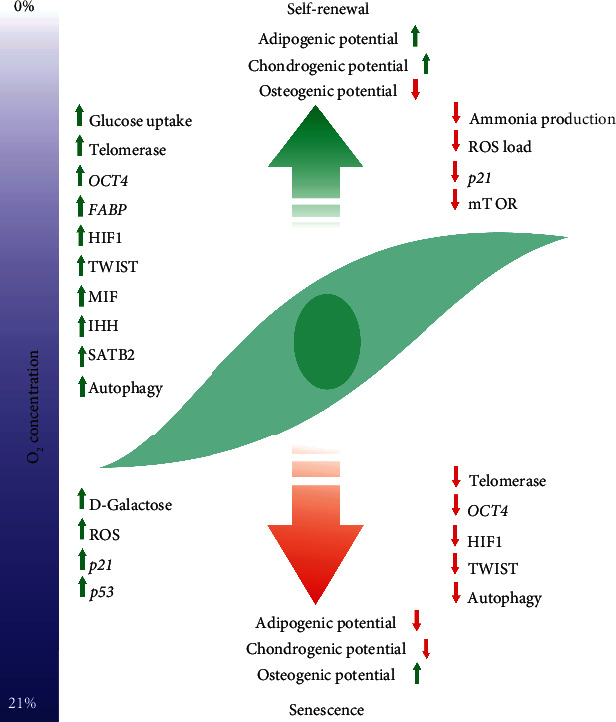
Schematic overview of the effects of hypoxia on BMSC senescence. Oxygen depletion induces or activates a number of cellular processes that delay or reverse cellular senescence in BMSCs. These include induction of stem cell-specific genes, upregulation of the HIF pathway and its effectors, or activation of autophagy. Parallel, hypoxia represses prosenescence factors like production of reactive oxygen species or expression of genes involved in cell cycle arrest and reduce activity of signaling cascades like the mTOR pathway. Abbreviations used are OCT4: octamer-binding protein 4; FABP3: fatty acid binding protein 3; TWIST: basic helix-loop-helix transcription factor; HIF1: hypoxia-inducible factor 1; IHH: Indian Hedgehog protein; MIF: macrophage inhibitory factor; SATB2: special AT-rich binding protein 2; mTOR: mammalian target of rapamycin; ROS: reactive oxygen species.

In cells exerting oxygen-dependent adenosine triphosphate (ATP) production, most of the oxygen is consumed by the oxidative phosphorylation. hBMSCs grown at 20% oxygen show a 2-fold increase in oxygen consumption in mitochondria compared to cells kept in hypoxic conditions that, in return, need to adjust their metabolism to adapt to oxygen depletion [49]. hBMSCs cultured at low oxygen tensions display elevated glucose uptake besides reduced incorporation of glucose-derived carbons in tricarboxylic acid cycle (TCA) intermediates indicating a complex metabolic switch in order to maximize energy production under anaerobic conditions (Figure 1.) [50]. In addition, hypoxic hBMSCs also display differential utilization of the carbon source glutamate in the TCA cycle. The accompanying low ammonia production, however, suggests that glutamate is primarily metabolized by transaminases rather than glutamate dehydrogenases in hypoxic hBMSCs [50]. Transamination does not lead to ammonia production upon the glutamate metabolism, and this may be beneficial to cells as ammonia is considered to be toxic to hBMSC cultures [51, 52]. Thus, the observation that hBMSCs grown at 2% oxygen show higher proliferation rates compared to hBMSCs kept at 20% oxygen raises the question if the differential proliferative capacity is, at least in part, due to the reduced ammonia production under hypoxic *in vitro* conditions [53]. Since proliferation often inhibits differentiation, one can speculate if hypoxia interferes with the differentiation capacity of BMSCs [54]. Indeed, the expression of telomerase reverse transcriptase (*TERT*) and octamer-binding protein 4 (*OCT4*), markers of undifferentiated cells, increases in hypoxic hBMSCs [55] (Figure 1). In addition, hypoxia seems to increase the subpopulation of G1-arrested hBMSCs cells that, apparently, fail to differentiate into bony or adipose tissues [56]. In contrast, however, other studies found elevated proliferation in both human and rodent hypoxic BMSC cultures accompanied by the repressed *OCT4* expression which may reflect the presence of distinct subpopulations in BMSC cultures that respond differentially to hypoxia [57, 58]. Indeed, cells that are more engaged toward the osteoblast lineage seem to behave differently than those more biased toward chondro- or adipogenesis. This concept is supported by findings that hypoxic hBMSC fail to display osteogenic characteristics such as calcification or alkaline phosphatase after 14 and 21 days of culture [49]. In accordanec, RUNX family transcription factor 2 (*RUNX2*), osteocalcin (*BGLAP*), and type I collagen (*COLI*) are all repressed in hypoxic hBMSCs while *VEGF* is induced suggesting that the HIF pathway is activated in these cells while the osteogenic machinery is not fully engaged [59] (Figure 1). These observations suggest that, for differentiation into osteogenic lineage, BMSCs need to be cultured at normoxic conditions. Interestingly, when hypoxic hBMSCs are transferred to normoxic conditions, cultures show osteogenic heterogeneity with differential osteogenic potential and suggesting that hypoxic adaptation induces permanent molecular changes that interfere with the, apparently, oxygen-demanding osteogenic programme [49]. In contrast, hypoxic hBMSCs show augmented adipogenic potential with induced adipocyte-specific genes and accumulation of lipid droplets compared to cells kept under normoxic culture conditions after 7-20 days [58, 60]. It is noteworthy, however, that there are also reports on attenuated adipogenesis under differential hypoxic conditions. Long-term hypoxia, for instance, apparently blocks adipogenic differentiation in hBMSC [61]. Although the exact reason of the higher oxygen demand of osteogenesis is not clear, it may be related to the decreased oxidative phosphorylation capacity of hypoxic BMSCs [49]. Alternatively, considering terminal differentiation as a special form of senescence, one can speculate if hypoxia interferes with the osteogenic differentiation of BMSCs by blocking their lineage-specific senescence.

### 1.6. BMSC Senescence and Low-Oxygen Conditions

There is a growing body of evidence that hypoxia affects BMSC senescence, at least in part by reprogramming cellular carbohydrate metabolism [49] (Table 1). Indeed, metabolites, like glyoxal, triggers hBMSC senescence which is accompanied by elevated levels of metabolic senescence markers including the senescence-associated *β*-galactosidase (SA-*β*-Gal) or *α*-L-fucosidase (*α*-Fuc) activity [62]. Moreover, metabolomics of the bone marrow of patients suffering from cyanotic congenital heart disease (CCHD) revealed enormously increased *D*-galactose levels provoking premature senescence of patient's BMSCs [63]. *D*-Galactose is a well-known senescence-inducing metabolite that acts, primarily, via oxidative stress [64]. Interestingly, elevated SA-*β*-Gal levels are long-recognized markers of senescence, so one can speculate that the role of *D*-galactose in BMSC senescence may not be necessarily restricted to severe chronic hypoxic conditions. Indeed, hBMSCs isolated from older, but not hypoxic donors also show elevated SA-*β*-Gal and lipofuscin levels parallel with reduced proliferative capacity compared to hBMSCs obtained from younger donors [65, 66]. Based on these findings, it seems that hypoxia has a pro=senescence effect in humans via senescence provoking metabolic changes (Figure 1).

The complex adaptive measures activated in oxygen-depleted environments, apparently, affect lipid metabolism as well. Indeed, the fatty acid binding protein 3 (FABP3), the muscle and heart-specific member of the fatty acid binding protein family, is upregulated in hypoxic hBMSCs [67]. FABP family members not only regulate the lipid metabolism and phospholipid biosynthesis, critical aspects of energy production and cell division, respectively, but also facilitate proliferation of endothelial cells and contribute to adipogenic differentiation as well [68–70]. Interestingly, however, hypoxia-induced FABP3 inhibits proliferation of hBMSCs that, in return, may reduce the accumulation of replicative mutations and, thus, senescence [67]. Indeed, in vitro proliferation of hBMSCs leads to accumulation of various mutations and chromosomal instabilities that eventually lead to cell cycle arrest and senescence [71]. Senescent BMSCs also accumulate reactive oxygen species (ROS) that, via oxidative DNA damage, not only reduce differentiation capacity but also upregulate p53 and p21, markers of senescence, leading to cell cycle arrest [72–76]. Senescent hBMSCs show elevated p53 and p21 activity and genetic ablation of p21 reverse senescent phenotype [77–79]. In addition, antioxidants or reduced ROS production slow premature senescence of hBMSCs and repress p21 and p53 expression [80, 81]. Thus, despite findings of the metabolic adaptation-triggered senescence, one can speculate that hypoxic conditions lead to an overall decrease of senescence by reducing ROS formation. Indeed, culturing BMSCs under the hypoxic condition has been reported to inhibit senescence and promote the undifferentiated status of cells [82, 83]. These phenotypic effects are preceded by HIF-mediated induction of the basic helix-loop-helix transcription factor TWIST (*TWIST1*) that results in consequent downregulation of p21 in hBMSCs [84]. In addition, in rodent cells, the antisenescence effect of HIF is, apparently, mediated by the HIF upregulated macrophage inhibitory factor (MIF), a known downregulator of p53 supporting the idea that the overall effect of hypoxia is rather anti-senescent and is mediated by the HIF pathway via multiple effectors [85] (Figure 1).

Similarly, contradicting results were obtained in studies analyzing the telomere length, another hallmark of senescence, of BMSCs under hypoxia. Cells with telomeres shorter than 10 kb usually show senescent phenotype including p53 and p21 activation and telomere aggregation that also leads to further DNA damage, and these hallmarks have been reported in senescent hBMSCs as well [86–88]. However, whether these changes are prevented or reverted by keeping BMSCs in the low oxygen environment is not clear as both elevated telomerase activities accompanied by increasing telomere length and telomere shortening have been observed in hypoxic hBMSC cultures [53, 84]. For the former one, the NAD-dependent protein deacetylase Sirtuin 1 (SIRT1) was reported to be involved in the maintenance of the telomerase activity and reduction of DNA damages at chromosomal ends in rat BMSCs (rBMSC). Indeed, inhibition of SIRT1 in young rBMSCs leads to premature senescence while its upregulation in old rBMSCs, apparently, decreases senescent phenotype and increases proliferation [89]. If SIRT1, however, is involved in the maintenance of telomeres under hypoxic conditions that is yet to be elucidated since, under hypoxia, intracellular NADH levels increase resulting in repression of S*IRT1* in established cell lines [90]. In addition, SIRT1 deacetylates HIF-1 and, thus, attenuates its transactivation activity, thereby counteracting HIF-mediated effects in hypoxic tubulointerstitial kidney cells [91]. Thus, the possible role of SIRT1 in the regulation of telomere length in hypoxic hBMSCs needs further investigations.

Besides the abovementioned metabolism-related mechanisms, autophagy also seems to be important in the maintenance of stem cell phenotype and inhibition of senescence [92]. Indeed, inhibition of autophagy by the autophagy inhibitor 3-methyladenine leads to early senescence of mBMSC. In contrast, activation of autophagy increases the proliferative capacity of the same cells [93]. Hypoxia, apparently, upregulates autophagy in hBMSCs by repression of the mTOR pathway, a known inhibitor of autophagy [94, 95]. Indeed, suppression of the mTOR pathway and ROS production by the Indian Hedgehog (IHH) protein reverses the senescent phenotype in hBMSCs including the downregulation of its own receptors PTCH1/2, one of the molecular hallmarks of aging hBMSCs [96, 97] (Figure 1). Another potentially autophagy-related regulator of BMSC senescence is the special AT-rich binding protein 2 (SATB2) that seems to be responsible for the elevated autophagy activity accompanied by higher pluripotency and anti-senescence capacity of mandibular hBMSC under both normoxic and hypoxic conditions. Activation of SATB2 leads to the increased expression of autophagy-related genes via the mTOR signaling pathway [98]. Furthermore, exogenous expression of SATB2 in tibial hBMSC leads to increased autophagic capacity and pluripotency [98].

## 2. Discussion

Since their low frequency in adult tissues requires *in vitro* expansion for cellular therapy purposes, understanding the mechanisms underlying BMSC senescence seems to be critical to the development of efficient BMSC-based therapeutical modalities. Still, our current knowledge on the effects of BMSC senescence is often controversial. Indeed, while secretion of proinflammatory agents by non-stem senescent cells favors M1 macrophage differentiation, senescent hBMSCs seem to retain anti-inflammatory properties by inhibiting both macrophages and lymphocyte proliferation [99–101]. Although this may suggest that BMSC senescence is not necessarily disadvantageous from the therapeutical point of view, it is now widely accepted that BMSC senescence fundamentally alters cellular characteristics so that it unfavorably affects their clinical potential.

Current data suggest that, during senescence, one of the affected aspects of cellular homeostasis is the carbohydrate metabolism. Data also suggest that, during oxygen depletion, the altered carbohydrate metabolism might contribute to the anti-senescence effect of hypoxia. Some data, however, suggest that certain aspects of the hypoxia-triggered metabolic alterations, like the increased *D*-galactose load, might also facilitate pro-senescence processes. Thus, these observations raise the question if the composition of the carbohydrate supply is critical in determining the overall effect of hypoxic preconditioning during *ex vivo* culturing of hBMSC.

Apparently, reactive oxygen species also play pivotal role in BMSC senescence so measures that reduce ROS production may also be considered effective *in vitro* interventions to prevent BMSC senescence. Indeed, reduction of the oxygen load by exposing cells to hypoxic environmental conditions seems to prolong BMSC lifespan and preserve their differentiation capacity toward, at least, the chondro- and adipogenic lineages. By the most plausible explanation, hypoxia reduces ROS formation that, consequently, leads to less molecular damage to cellular components. This idea is, apparently, supported by observations that mechanisms that are involved in the elimination of damaged cellular components, like autophagy, can revert the senescent phenotype. In accordance, besides lysosome-mediated autophagy, the proteasome system also seems to be involved in the control of BMSC senescence. Indeed, downregulation of ubiquitin C *(UBC)* leads to replicative senescence in BMSC the overexpression of *UBC* in hBMSC leads to increased proliferation and repression of senescence [102]. Unfortunately, no significant data was obtained on *UBC* expression under different oxygen conditions to date so determination of the potential role of ubiquitination in BMSCs senescence under hypoxia needs further investigation.

Although the use of the low oxygen environment for *ex vivo* culturing of BMSCs seems logical, conflicting observations on the behavior of BMSC cultures under *in vitro* hypoxic conditions indicate the need of further investigation to detail the optimal use of hypoxia for BMSC expansion. These investigations, however, need to be standardized since current data indicate species-specific BMSC responses to hypoxia. Indeed, while rBMSCs show increased proliferation and expression of osteogenic lineage markers, like the alkaline phosphatase activity and accumulation of calcium under hypoxic culture conditions, hBMSCs show paralyzed osteogenic differentiation in oxygen-depleted cultures [103, 104]. Similarly, senescence achieved by hBMSCs due to shortened telomeres is irreversible while rat BMSCs can escape the telomere shortening-mediated senescence and become malignant [105].

The mode of application of hypoxia to *ex vivo* expanded BMSCs needs further characterization as well. Short-term hypoxia seems to be beneficial in terms of BMSC senescence while long-term pathological hypoxia induces premature senescence in human BMSCs. Indeed, BMSCs from patients with CCHD were predisposed to premature senescence and had impaired multi-lineage differentiation potential [63]. Beside duration, the mode of exposure also seems to be critical. Hypoxic pre-conditioning of rBMSCs for 7 days followed by 7 day-long exposure to ambient oxygen conditions results in increased proliferation, augmented osteogenic differentiation, and induction of the anti-apoptotic B-cell CLL/lymphoma 2 (*BCL2*) and Bcl-2-like protein 1 encoding genes (*BCL2L1*) compared to cultures kept under either ambient air or hypoxia continuously for 14 days [106].

Some data, however, suggest that even hypoxia is not capable to reverse all aspects of senescence. BMSCs isolated from elderly patients suffering from osteoarthritis failed to show robust differentiation into osteo-, chondro-, or adipocyte lineages under hypoxic conditions. These BMSCs display reduced expression of cell lineage-specific genes like the SRY-box transcription factor 9 (*SOX9*) and collagen type I alpha 1 chain (*COL1A1*) for chondrocyte lineage, alkaline phosphatase (*ALPL*), and osteogenic protein (*OP*) for osteocyte lineage and *BGLAP* and fatty acid binding protein 4 (*FABP4*) for adipocyte lineage compared to cells from the same isolates kept under ambient oxygen tensions indicating the potential pro-senescence effect of the chronic inflammatory milieu and/or exhausted adaptive measures to cope with hypoxia [107]. In addition, premature senescence of BMSCs of CCHD patients seems to be mediated by gut microbiota dysbiosis, especially by reduction in Lactobacillus, via systemic deviation in carbohydrate metabolism indicating that even, apparently, irrelevant underlying medical conditions may affect the quality of isolated BMSCs [63].

## 3. Conclusion

Despite the occasionally conflicting observations on the effects of oxygen depletion on BMSC senescence, hypoxic exposure of human BMSCs is now believed to be beneficial for delaying permanent cell cycle arrest of *ex vivo* cultured BMSCs. However, further investigations are needed to define detailed protocols that minimize the potentially disadvantageous effects of hypoxic preconditioning while maximizing its anti-senescent ones. These works, at the same time, will not only help to understand the role of oxygen in cellular senescence but shed light on the underlying molecular mechanism leading us closer to the fundamentals of BMSC physiology.

## Figures and Tables

**Table 1:** Summary of studies investigating the effects of hypoxia on BMSC senescence.

Study findings	References
Proliferation and cellular metabolism of hypoxic human BMSCs	[47, 50, 53, 56, 58, 63, 67, 82–84]
Proliferation and osteogenic capacity of hypoxic rat BMSCs	[103]
Osteogenic differentiation of hypoxic human BMSCs	[49, 55, 56, 58, 61, 63, 82, 104, 106, 107]
Adipogenic differentiation of hypoxic human BMSCs	[56, 58, 60, 61, 63, 82, 107]
Chondrogenic capacity of hypoxic human BMSCs	[63, 82, 107]
Chondrogenic capacity of hypoxic rat BMSCs	[57]
Autophagy in hypoxic human BMSCs	[94]

## Abbreviations

ATP: Adenosine triphosphate
CCHD: Cyanotic congenital heart disease
CCR7: Chemokine receptor 7
CXCR4: Chemokine receptor type 4
EPO: Erythropoietin
FABP3: Fatty acid binding protein 3
hBMSCs: Human BMSCs
HIF: Hypoxia-inducible factor
HIF*α*: Hypoxia-inducible factor-alpha
HIF*β*: Hypoxia-inducible factor-beta
HIF1: Hypoxia-inducible factor 1
IHH: Indian hedgehog
IL: Interleukin
mBMSC: Mouse BMSCs
M-CSF: Macrophage colony-stimulating factor
MIF: Macrophage inhibitory factor
OCT4: Octamer-binding protein 4
PHDs: Prolyl hydroxylases
pVHL: Von Hippel-Lindau
rBMSC: Rat BMSCs
ROS: Reactive oxygen species
RUNX2: RUNX family transcription factor 2
SATB2: Special AT-rich binding protein 2
SA-*β*-Gal: Senescence-associated *β*-galactosidase
SCF: Stem cell factor
SASP: Senescence-associated secretory phenotype
SIRT1: Sirtuin 1
TWIST: Basic helix-loop-helix transcription factor
TERT: Telomerase reverse transcriptase
TCA: Tricarboxylic acid cycle
UBC: Ubiquitin C
VEGF: Vascular endothelial growth factor
*α*-Fuc: *α*-L-fucosidase.
